# Sexual function and sexual quality of life in men with genital warts: a cross-sectional study

**DOI:** 10.1186/s12978-022-01403-z

**Published:** 2022-04-27

**Authors:** Marzieh Hosseini Nia, Fatemeh Rahmanian, Mehdi Ghahartars, Roksana Janghorban

**Affiliations:** 1 Student Research Committee, Department of Midwifery, School of Nursing and Midwifery, Shiraz University of Medical Sciences, Shiraz, Iran; 2 Department of Midwifery, School of Nursing and Midwifery, Community Based Psychiatric Care Research Center, Shiraz University of Medical Sciences, Shiraz, Iran; 3 Molecular Dermatology Research Center, Shiraz University of Medical Sciences, Shiraz, Iran

**Keywords:** Genital warts, Human papilloma virus, Sex, Sexual dysfunction, Quality of life

## Abstract

**Background:**

Human papillomavirus is the most common sexually transmitted infection, usually passing from one person to another after the first sexual activity. Infection with this virus and the occurrence of genital warts (GWs) could have several effects on patients’ health. This study was performed to evaluate the sexual function and sexual quality of life (SQOL) in men with GWs.

**Methods:**

This cross-sectional study was conducted from September 2019 to March 2020 with a sample size of 105 men with GWs in the dermatology clinic of Shahid Faghihi Hospital in Shiraz University of Medical Sciences, Iran. Data were collected using a demographic questionnaire, the International Index of Erectile Function (IIEF), and the Sexual Quality of Life-Men (SQOL-M) questionnaires and analyzed by descriptive and inferential tests with SPSS software version 22.

**Results:**

The mean score for overall sexual function in men was 48.50 ± 8.89. About 35.2% of men had overall sexual dysfunction (SD). The highest disorder rate was related to the erection domain (85.7%), and the lowest was related to the desire domain (5.7%). In the erection domain, most men (54.3%) experienced mild to moderate erectile dysfunction. The mean score for SQOL-M was 38.36 ± 14.47, and 56.2% of them had a good SQOL.

**Conclusions:**

GWs affected men’s erection more than the other sexual function domains. SD in men with GWs has a significant impact on their SQOL and ED was associated with impaired SQOL. Therefore, it is recommended to pay more attention to SD screening alongside SQOL assessment of men with GW.

## Plain language summary

Human papillomavirus is the most common sexually transmitted infection. Infection with this virus and the occurrence of genital warts (GWs) could have several effects on men and women’s health. In this cross-sectional study, we examined the sexual function and sexual quality of life (SQOL) in men with GWs. Of the 105 men, 35.2% had sexual dysfunction (SD). The highest disorder rate was related to the erection domain (85.7%), and the lowest was related to the desire domain (5.7%). In the erection domain, most men (54.3%) experienced mild to moderate erectile dysfunction. 56.2% of them had a good SQOL. SD in men with GWs has a significant impact on their SQOL. Therefore, it is recommended that assessment of sexual function and SQOL should be done when men with GWs refer to health services.

## Background

Genital human papillomavirus (HPV) infection is the most common sexually transmitted infections (STIs), usually passing from one person to another after the first sexual activity [1]. According to the World Health Organization, in 2019, about 600 million men and women worldwide were infected by the virus [2]. Although most studies have focused on women and the association of HPV infection with cervical cancer, the prevalence of the virus in men is also significant. The results of the National Health and Nutrition Examination Surveys between 2013 and 2014 in the United States showed that 42.2% of American men aged from 14 to 59 years have some form of genital HPV infections, and 2.23% of men in this age group reported a history of genital warts (GWs) [3, 4]. HPV and related diseases are not specific to women, and Genital HPV can develop as GWs in both women and men. In Commercially Insured US Populations, the incidence of GWs was 1.2 cases per PY 1000 among women and 1.1 cases per PY 1000 among men, with the highest incidence among women aged from 20 to 24 years and men 25 to 29 years [5].

Several studies have reported GWs morbidity as a psychosocial burden, including worries, emotional and sexual effects, self-image concern, and control/life impact. However, women experienced more Burden than men. Men were also affected by the psychological effects of GWs [6, 7].

In the literature, it has been shown that STIs have negative effects on men’s mental health and sexual function [8–10]. Some studies also indicate sexual-related concern in men with GWs [6, 7]. Because the sexual response cycle reflects biopsychosocial interaction, these concerns appear to affect sexual function, but there is limited evidence [11].

One of the most critical issues in sexual health is the quality of sexual life. Previous studies have shown that GWs in men are associated with severe damage to their health-related quality of life [7, 12, 13]. Based on the available shreds of evidence, no studies have focused on Sexual Quality of Life-Men (SQOL-M) with GWs. Therefore, this study was designed to evaluate the sexual function and SQOL in men with GWs.

## Methodology

### Study design

This cross-sectional study was conducted from September 2019 to March 2020 in the dermatology clinic of Shahid Faghihi Hospital at Shiraz University of Medical Sciences, Iran, among men with GWs.

### Study population

The study included men whose GWs were diagnosed by a dermatologist based on clinical signs in their skin. The sample size was determined by considering a confidence interval of 95%, the study power of 90% and according to data of previous study with an effect size of 0.35 [11]. The minimum sample size of 87 was obtained; also, considering 20% of the sample loss, 105 samples were considered. Inclusion criteria included: 18 years and above (cut off age for adult men), having sex in the last 4 weeks, absence of history or current of mental illness under treatment, no use of drugs affecting sexual function (antihypertensive drugs, cardiovascular drugs, antidepressants, opiates, blood sugar regulators and amphetamines), and no chronic diseases such as diabetes, hypertension, autoimmune diseases, and heart disease. The exclusion criterion was withdrawal from the company after enrollment.

### Data collection

Sociodemographic information and data about sexual orientation and sexual style were collected by a researcher-made questionnaire. Sexual function was assessed by the International Index of Erectile Function (IIEF), a self-report questionnaire. The questionnaire consists of 15 items, each of which is graded on a 5- or 6-point Likert-type response scale and examines five domains: erection, orgasm, desire, intercourse satisfaction, and overall satisfaction. A higher score indicates better sexual function, and the maximum acceptable score of 75 indicates the best sexual status in various areas [14, 15]. The status of patients’ erectile function was also considered as follows: 0–10 (severe erectile dysfunction (ED)), 11–16 (moderate ED), 17–21 (moderate to mild ED), 22–25 (mild ED), 26–30 (no ED) [16].

The SQOL-M was assessed using the 11-item questionnaire. The total score is between 11 and 66, and a higher score indicates a better SQOL-M [17]. A score of 11–22 was considered a poor class, a score of 23–33 was considered a middle class, and a score of 34 and above was considered a good class [18].

### Ethical issues

The research project was approved by the ethics committee of Shiraz University of Medical Sciences (IR.SUMS.REC.1398.743). Written informed consent was obtained from all the participants.

### Data analysis

The data were summarized using frequencies and percentages. Continuous variables were summarized as the mean and standard deviation. Categorical variables were compared using the χ^2^ test or Fisher’s exact test. Correlations between variables were determined using Spearman and Pearson correlation coefficient. logistic regression analysis was used to evaluate the association between ED and SQOL-M. The data were analyzed by SPSS software version 22 (SPSS Inc., Chicago, IL, USA). P value < 0.05 was considered significant.

## Results

A total of 105 men completed the study. The mean age of men was 30.86 ± 7.3 years, and the mean duration of GWs was 19.92 ± 20.45 months. Table 1 shows some demographic characteristics and their sexual relationships (Table 1).

**Table 1:** Baseline characteristics of participants

Variables	Number (%) (n = 105)
Education	
Junior high school and below	7 (6.66)
Senior high school	36 (34.28)
College and above	62 (59.04)
Marital status	
Permanent marriage	48 (45.7)
Temporary marriage	3 (2.9)
Single with partner	54 (51.4)
Number of intercourse in week	
1	26 (24.8)
2	41 (39)
≥ 3	38 (36.1)
Extramarital relationship in married people	
Yes	17 (33.3)
No	34 (66.6)
Number of partner	
1	68 (64.8)
2	19 (18.1))
3	9 (8.6)
≥ 4	9 (8.6)
Sexual orientation	
Homosexual	4 (3.8)
Heterosexual	101 (96.1)
Bisexual	0
Sexual style	
Vaginal	45 (42.9)
Rectal	4 (3.8)
Oral	2 (1.9)
Vaginal and oral	21 (20)
Vaginal and anal	1 (1)
Vaginal, oral, and anal	32 (30.5)
Condom use in intercourse	
Yes	86 (81.9)
No	19 (18.1)

According to Tables 2 and 3, the mean score for overall sexual function in men was 48.50 ± 8.89, and according to the subscales of the IIEF Questionnaire, 35.2% of men had general dysfunction. Among the different domains of this disorder, the highest rate of disorder was related to the erectile domain (85.7%), and the lowest rate was related to the desire domain (5.7%).

**Table 2:** Mean score of sexual function and subscales measured by the International Index of Erectile Function (IIEF) score in men with genital warts

IIEF domains						
Measure	Erectile function	Orgasmic function	Sexual desire	Intercourse satisfaction	Overall satisfaction	Total score
Mean	20.22	6.46	6.76	8.67	6.37	48.50
SD	4.041	1.88	1.59	2.03	1.84	8.89
SE	0.394	0.184	0.155	0.198	0.179	0.868
Median	20	6	6	9	6	48

**Table 3:** Frequency of sexual dysfunction measured by the International Index of Erectile Function (IIEF) score in men with genital warts

IIEF domains						
	Erectile function	Orgasmic function	Sexual desire	Intercourse satisfaction	Overall satisfaction	Total score
Cut of point	≤ 12	≤ 4	≤ 4	≤ 6	≤ 4	≤ 30
N (%)	90 (85.7)	20 (19)	6 (5.7)	15 (14.3)	21 (20)	37 (35.2)

Table 4 shows that in the area of erection, most men (54.3%) experienced mild to moderate ED. The total score of IIEF and domains did not correlate with age, time since onset of genital warts, and education. The mean total score of SQOL-M in this study was 38.36 ± 14.47, and Table 4 showed that 56.2% of individuals had a score of 34 and above and were classified as the good SQOL.

**Table 4:** Severity of erectile dysfunction (ED) and Sexual quality of life status measured by the Sexual Quality of Life-Men (SQOL-M) in men with genital warts

	N (%)	P value
*ED severity types*		
Severe	0	0.001
Moderate	17 (16.2)	
Moderate to mild	57 (54.3)	
Mild	16 (15.2)	
No ED	15 (14.3)	
*Sexual quality of life status*		
Poor	15 (14.3)	0.001
Middle	31 (29.5)	
Good	59 (56.2)	

The SQOL-M scores correlated significantly with education and all IIEF domains but not with age and time since the onset of GWs (Table 5).

**Table 5:** Correlations of sexual quality of life (SQOL-M score) with age, time since onset of genital warts, and the International Index of Erectile Function (IIEF) domains

Independent variable	Correlation with SQoL-M score	P value
Age	0.01	0.22
Education	0.27	0.004
Time since onset of genital warts	0.05	0.54
IIEF desire	0.247	0.001
IIEF erectile function	0.489	0.001
IIEF orgasmic function	0.558	0.001
IIEF intercourse satisfaction	0.45	0.001
IIEF overall satisfaction	0.374	0.001
IIEF total score	0.565	0.001

Logestic regression analysis revealed that ED in men was associated with 61% increase in odds of impaired SQOL (OR:1.61, 95% CI:0.09–1.08, P-value:0.023) (Table 6).

**Table 6:** Results of logistic regression associated with sexual quality of life

Variable	β	Standard error	P value	Odds ratio (95% confidence interval for EXP(B))
Age	0.05	0.04	0.222	1.05 (0.97–1.14)
Education	0.02	0.16	0.903	1.02 (0.73–1.41)
Marital status	− 0.53	0.53	0.325	0.58 (0.20–1.69)
Time since onset of genital warts	− 0.09	0.01	0.464	0.99 (0.96–1.01)
IIEF* desire	0.11	0.07	0.150	1.11 (0.96–1.30)
IIEF erectile function	0.47	0.21	0.023	1.61 (1.06–2.43)
IIEF orgasmic function	− 0.32	0.22	0.136	0.721 (0.46–1.10)
IIEF intercourse satisfaction	0.05	0.15	0.735	1.05 (0.78–1.42)
IIEF overall satisfaction	0.12	0.15	0.420	1.12 (0.84–1.51)

*International Index of Erectile Function (IIEF)

## Discussion

More than one-third of men with GWs had impaired overall sexual function in this study, and ED was the most common disorder. The prevalence of ED in this group was considerably higher than the pooled prevalence estimation in the general population from a meta-analysis of 11 studies (56.1%, 95% confidence interval [CI]: 40–72%) [19]. In addition, the prevalence of mild to moderate ED in the present study was higher than that of Asian men: 5 to 31.6% vs. 54.3% [20]. Prevalence of sexual dysfunction increases in certain conditions such as chronic diseases. Evaluation of ED using IIEF in some chronic diseases of Iranian men such as congestive heart failures, hemodialysis, diabetes mellitus type II showed that the prevalence of this disorder was about 80%, which was close to that found in men with GWs [19]. Therefore, it seems that GWs should be considered a condition that can negatively impact male sexual function, especially erectile function. The presence of ED in men with GWs is consistent with the study of Kucukunal et al. In this study, sexual dysfunction was also more common in men with GWs compared to the control group, and these men experienced more abnormalities in sex drive, arousal, penile erection, ability to reach orgasm and satisfaction from orgasm [11].

Regarding the study of the sexual function of these men, the El-Hamd study aimed to assess premature ejaculation (PE) frequency and intravaginal ejaculatory latency time. The results showed that 66.7% of men with GWs had PE. However, the rate of anxiety and PE in patients with GWs and PE disorder was higher and significantly higher than men who had GWs but did not have PE disorder compared to the control group (P = 0.001) [21].

The presence of sexual disorders, especially ED, in men with other STIs such as human immunodeficiency virus (HIV) and Herpes simplex virus (HSV) has also been reported, like HPV [8, 10]. In these diseases, in addition to atherosclerotic changes and problems with the organic vascular component sexual function that occurs in some diseases such as HSV, seems to cause psychological distress and emotional concerns about the possibility of partner involvement and the appearance of skin lesions, which can lead to the high attention to sexual performance during intercourse and lead to ED [8, 10].

Several studies have reported an association between age and ED. But most of these studies have focused on older men or men whose average age was over 40 years [22–25]. The absence of such an association in our study appears to be due to the low average age (approximately 30 years) of men with GWs in this study.

Published studies have reported different findings of the quality of life of people with GWs. To date, no study has focused specifically on the SQOL of people with GWs. Some of these studies suggest mild or no impairment of quality of life in men [26, 27], and some reported that the quality of life in people (men and women) is significantly lower [12, 28]. However, studies have shown that the effects of psychosocial GWs on the health-related quality of life of women are more significant than men [12, 29]. More than half of the men had a good SQOL in the present study, and about one-third had a moderate SQOL.

In the present study, sexual dysfunction had a noticeable impact on the SQOL and ED was associated with increase in odds of impaired SQOL. The study conducted by Owiredu et al. on men and women with physical disabilities showed that sexual dysfunction was higher in this group than healthy individuals, and this dysfunction significantly affected their SQOL [30]. This effect was also confirmed in men with GWs and without physical disability in the present study. A review article showed that men with ED have a poor quality of life and even more economic burden than men without ED [31]. The findings confirmed with Van Vo et al. study that one-third of the married men with ED reported having low quality of life [32]. Although, the result focused on general quality of life of men with ED, some studies revealed that ED adversely affects the relationship with a partner and decreased sexual satisfaction in their female partners [33–35]. It seems that ED, in addition to negatively impact on female partners’ sexual life, can also impose a substantial SQOL burden in men. The issue is consistent with the results of the current study.

Some potential limitations should be discussed. Nearly all men in this study were heterosexual. Therefore the results are not generalized in men with GWs with different sexual orientations. This study did not consider the severity of GWs, men’s stress and anxiety level, and sexual partner status in terms of suffering from GWs. Future studies should consider these variables, which may affect sexual function and SQOL.

## Conclusions

The results showed that more than one-third of men with GWs had impaired overall sexual function, and most had ED. More than half of the men had a good SQOL, about a third had a moderate SQOL, and the level of sexual dysfunction clearly impacted the SQOL. ED was associated with impaired SQOL.These results could have an important application in a comprehensive and tailored view of the needs of men with GWs. Healthcare providers should pay more attention to the sexual function, and SQOL in men with GWs, as these can affect the sexual health of men and their sexual partners.

## Data Availability

The datasets used are available from the corresponding author upon reasonable request.

## Abbreviations

ED: Erectile dysfunction
GWs: Genital warts
HIV: Human immunodeficiency virus
HPV: Human papillomavirus
HSV: Herpes simplex virus
IIEF: International Index of Erectile Function
PE: Premature ejaculation
SD: Sexual dysfunction
SQOL: Sexual quality of life
SQOL-M: Sexual Quality of Life-Men
STIs: Sexually transmitted infections
